# Supplementary material for: Selection on heritable social network positions is context-dependent in Drosophila melanogaster
Source: Nat Commun. 2021 Jun 7;12:3357. doi: 10.1038/s41467-021-23672-1 (PMC8185000; doi:10.1038/s41467-021-23672-1)
Supplement: Supplementary file 1 — Supplementary Information [file 41467_2021_23672_MOESM1_ESM.pdf]

Selection on heritable social network positions is context-dependent in *Drosophila melanogaster* (Supplementary Figures and Tables)

Eric Wesley Wice[1]* and Julia Barbara Saltz[1]

[1] Department of Biosciences, Rice University, Houston, TX 77005

*Corresponding author (eric.wesley.wice@gmail.com)

SUPPLEMENTARY FIGURE 1

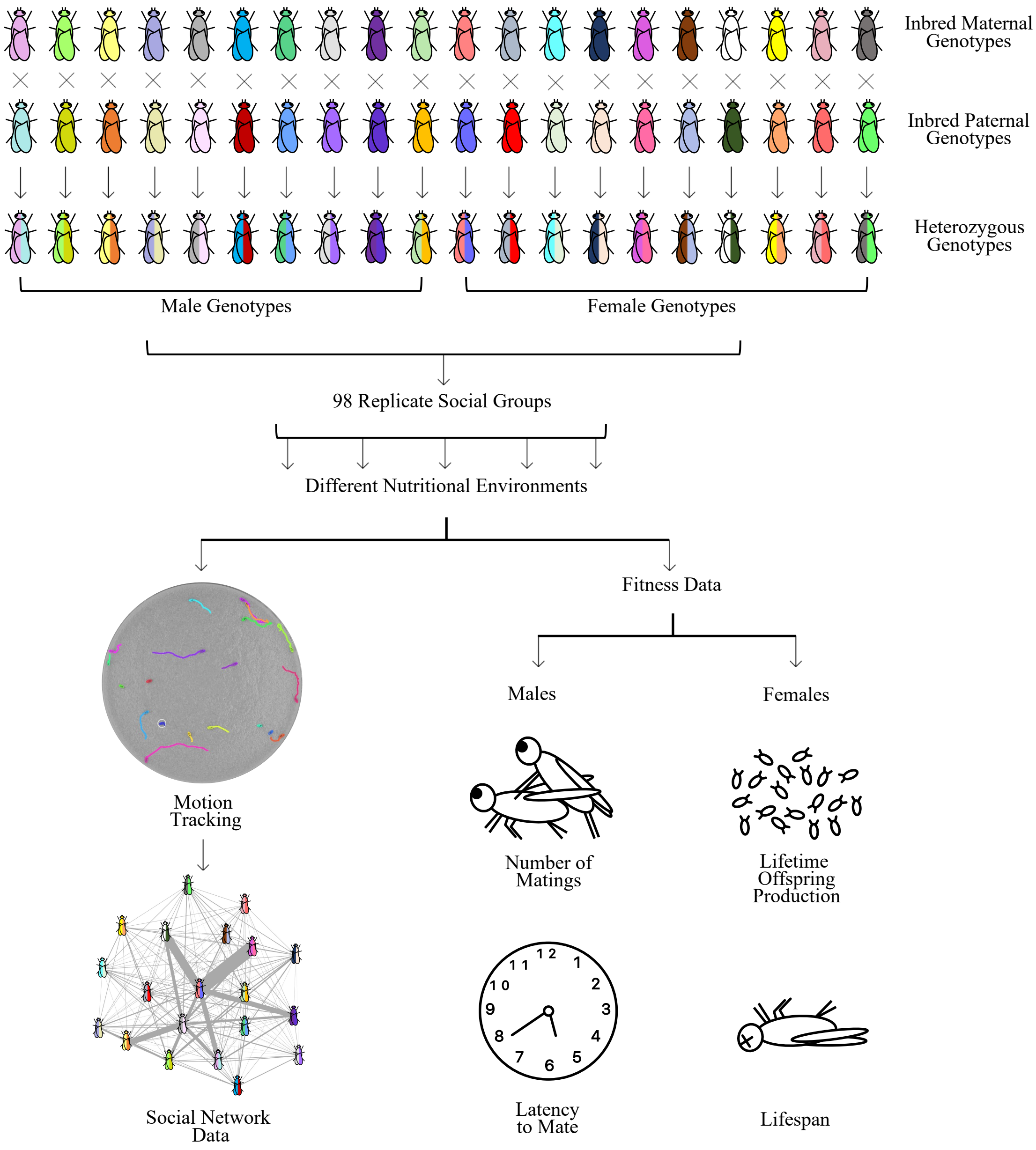

Supplementary Figure 1 | Experimental schematic. Solid-colored flies represent the 40 DGRP inbred lines that were uniquely crossed to create 20 heterozygous genotypes (bi-colored flies). The 20 heterozygous genotypes were combined into a social group, which was replicated 98 times. Each social group was placed on one of five nutritional environments. The motion-tracking and social network data presented are representative of data gleaned from a single social group.

SUPPLEMENTARY FIGURE 2

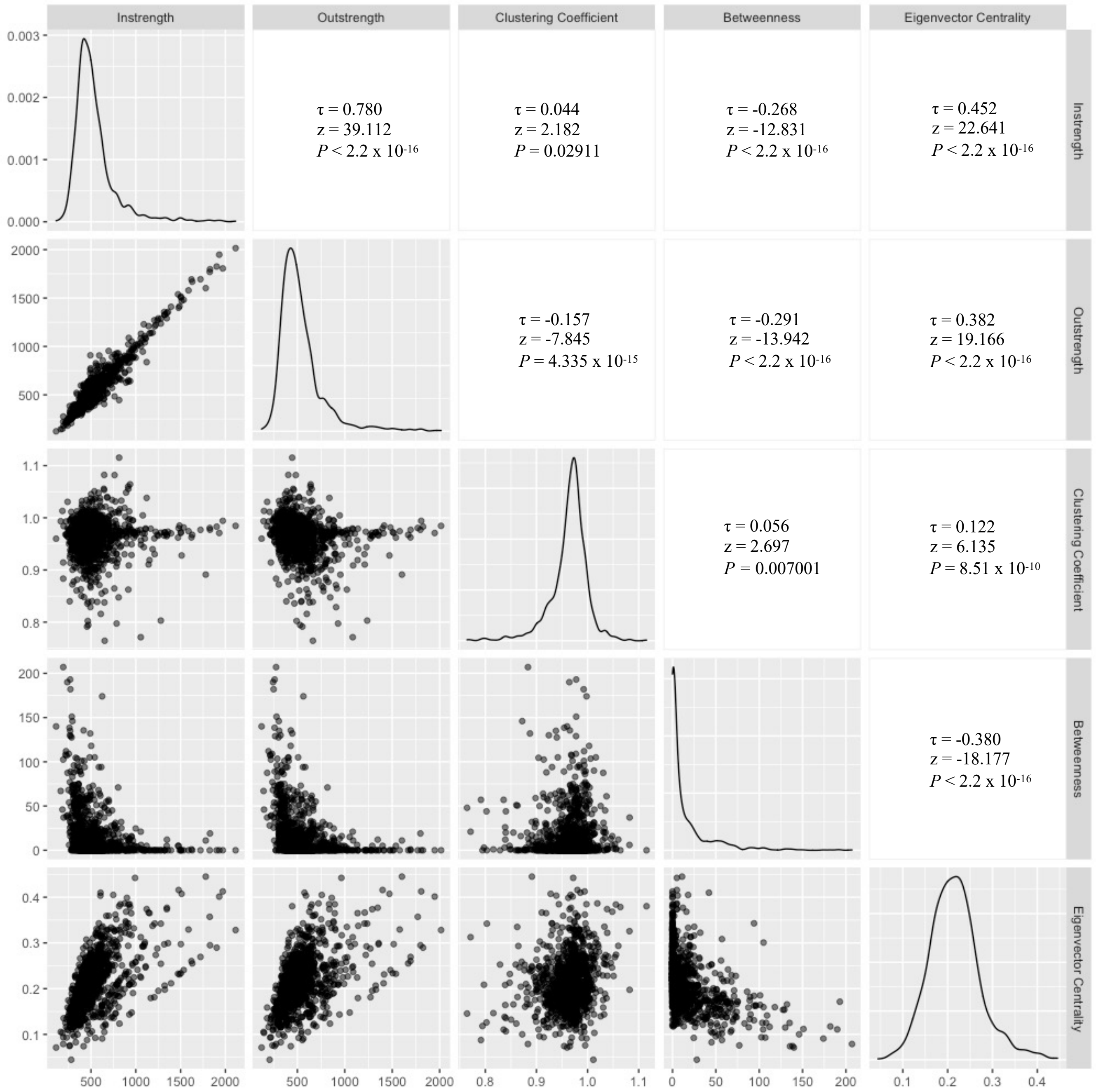

Supplementary Figure 2 | Correlations between network position variables. Diagonal shows the distributions of each of the five network variables. Lower-left scatterplots show plotted relationships between all pairwise combinations of network variables. Upper-right quadrants show two-sided Kendall's rank correlation estimates for each pairwise combination of network variables. Source data are provided as a Source Data file.

SUPPLEMENTARY TABLE 1

Social Network Positions	Sex				Genotype				Nutritional Environment<br>P:C Ratio<br>Caloric Concentration			Sex-by-Environment<br>Sex-by-P:C Ratio<br>Sex-by-Caloric Concentration				Genotype-by-Environment<br>Genotype-by-P:C Ratio<br>Genotype-by-Caloric Concentration			
	χ^2	Df	*P*	P_R	LRT	Df	*P*	P_R	χ^2	Df	*P*	χ^2	Df	*P*	P_R	LRT	Df	*P*	P_R
									6.306	4	0.177	4.184	4	0.382	0.287	4.284	14	0.994	0.195
Instrength	8.072	1	0.004	**0.002**	22.713	1	<0.001	**<0.001**	5.175	1	**0.023**	0.904	1	0.342	0.301	0	2	1	0.591
									1.130	1	0.288	0.014	1	0.908	0.907	1.260	2	0.533	0.082*
									9.492	4	**<0.050**	13.691	4	0.008	**0.002**	1.026	14	1	0.543
Outstrength	10.108	1	0.001	**0.001**	24.13	1	<0.001	**<0.001**	8.122	1	**0.004**	9.599	1	0.002	**<0.001**	0	2	1	0.512
									2.462	1	0.117	4.591	1	0.032	**0.033**	0.820	2	0.664	0.109
									9.873	4	**0.043**	38.130	4	<0.001	**<0.001**	5.000	14	0.986	**0.007**
Clustering Coefficient	106.832	1	<0.001	**<0.001**	54.726	1	<0.001	**<0.001**	7.057	1	**0.008**	25.890	1	<0.001	**<0.001**	1.843	2	0.398	**0.001**
									11.618	1	**<0.001**	33.250	1	<0.001	**<0.001**	1.047	2	0.592	**0.028**
									2.267	4	0.687	55.956	4	<0.001	0.885	2133.3	14	<0.001	0.853
Betweenness Centrality	6.208	1	0.013	0.683	1086	1	<0.001	**0.022**	0.582	1	0.446	13.761	1	<0.001	0.451	625.07	2	<0.001	0.368
									0.341	1	0.559	11.432	1	<0.001	0.626	565.97	2	<0.001	0.782
									8.334	4	0.080*	4.778	4	0.311	0.319	0	14	1	0.997
Eigenvector Centrality	9.248	1	0.002	**0.006**	24.348	1	<0.001	**<0.001**	0.147	2	0.929	0.773	1	0.379	0.390	0.236	2	0.889	0.447
									8.097	2	**0.017**	0.208	1	0.648	0.621	0.537	2	0.765	0.251

Supplementary Table 1 | Model results for effects on network positions. Both observed p-values (*P*), and p-values gleaned from one-sided network permutation tests (P_R) are presented. Bolded P_R values indicate significant effects (* indicates marginal significance). Source data are provided as a Source Data file.

SUPPLEMENTARY TABLE 2

Social Network Positions	Sex				Genotype				Nutritional Environment			Sex-by-Environment				Genotype-by-Environment				Activity			
	χ^2	Df	*P*	P_R	LRT	Df	*P*	P_R	χ^2	Df	*P*	χ^2	Df	*P*	P_R	LRT	Df	*P*	P_R	χ^2	Df	*P*	P_R
Instrength	4.815	1	0.028	**0.015**	25.339	1	<0.001	**<0.001**	6.166	4	0.187	3.504	4	0.477	0.380	4.231	14	0.994	0.206	4.624	1	0.032	0.570
Outstrength	11.948	1	<0.001	**0.001**	26.814	1	<0.001	**<0.001**	9.314	4	0.054*	12.598	4	0.013	**0.003**	1.052	14	1	0.555	5.001	1	0.025	0.440
Clustering Coefficient	114.125	1	<0.001	**<0.001**	57.175	1	<0.001	**<0.001**	9.930	4	**0.042**	36.711	4	<0.001	**<0.001**	3.958	14	0.996	**0.016**	13.601	1	<0.001	0.348
Betweenness Centrality	0.654	1	0.419	0.888	984.26	1	<0.001	**0.012**	12.829	4	**0.012**	56.058	4	<0.001	0.798	1775.9	14	<0.001	0.819	2774.847	1	<0.001	**<0.001**
Eigenvector Centrality	8.798	1	0.003	**0.002**	24.236	1	<0.001	**<0.001**	8.334	4	0.080*	4.752	4	0.314	0.291	0	14	1	0.997	0.006	1	0.938	0.961

Supplementary Table 2 | Model results for effects on network positions with activity covariate. Both observed p-values (*P*), and p-values gleaned from one-sided network permutation tests (P_R) are presented. Bolded P_R values indicate significant effects (* indicates marginal significance). Source data are provided as a Source Data file.

SUPPLEMENTARY TABLE 3

Fitness Effects	Number of Matings			Latency to Mate			Lifetime Offspring Production			Lifespan		
	Test Statistic	Df	*P*	Test Statistic	Df	*P*	Test Statistic	Df	*P*	Test Statistic	Df	*P*
Genotype	99.76	1	**<0.001**	87.104	1	**<0.001**	0.04	1	0.842	0.16	1	0.689
Nutritional Environment	1.021	4	0.907	2.418	4	0.659	6.84	4	0.145	27.277	4	**<0.001**
P:C Ratio	0.273	1	0.601	1.100	1	0.294	4.860	1	**0.028**	7.443	1	**0.006**
Caloric Concentration	0.113	1	0.737	0.020	1	0.886	<0.001	1	0.989	10.815	1	**0.001**
Instrength	8.795	1	**0.003**	9.687	1	**0.002**	0.857	1	0.355	0.007	1	0.936
Outstrength	4.075	1	0.044^	3.070	1	0.080^{+}	0.570	1	0.450	0.015	1	0.903
Clustering Coefficient	2.410	1	0.121	3.814	1	0.051^{+}	0.717	1	0.397	0.000	1	0.996
Betweenness Centrality	2.080	1	0.149	0.505	1	0.478	1.593	1	0.207	2.138	1	0.144
Eigenvector Centrality	9.938	1	**0.002**	0.009	1	0.925	1.406	1	0.439	1.150	1	0.284
Instrength-by-Environment	52.014	4	**<0.001**	13.720	4	**0.008**	7.074	4	0.132	1.017	4	0.907
Instrength-by-P:C Ratio	6.478	1	0.011*^	7.317	1	**0.007**	4.130	1	0.042^	0.816	1	0.366
Instrength-by-Caloric Concentration	7.098	1	**0.008**	10.367	1	**0.001**	2.002	1	0.157	0.345	1	0.557
Outstrength-by-Environment	20.440	4	**<0.001**	10.602	4	0.031^	4.130	4	0.389	2.184	4	0.702
Outstrength-by-P:C Ratio	5.733	1	0.017*^	8.183	1	**0.004**	2.341	1	0.126	1.540	1	0.215
Outstrength-by-Caloric Concentration	2.699	1	0.100	6.471	1	0.011*^	1.311	1	0.252	0.853	1	0.356
Clustering Coefficient-by-Environment	5.875	4	0.209	4.128	4	0.389	0.883	4	0.927	1.220	4	0.875
Clustering Coefficient-by-P:C Ratio	0.019	1	0.890	0.057	1	0.811	0.502	1	0.479	0.402	1	0.526
Clustering Coefficient-by-Caloric Concentration	1.676	1	0.196	2.293	1	0.130	<0.001	1	0.993	0.813	1	0.367
Betweenness Centrality-by-Environment	7.473	4	0.112	5.857	4	0.210	2.943	4	0.567	5.371	4	0.251
Betweenness Centrality-by-P:C Ratio	2.117	1	0.146	0.395	1	0.530	1.359	1	0.244	3.471	1	0.063^{+}
Betweenness Centrality-by-Caloric Concentration	0.516	1	0.473	2.910	1	0.088^{+}	0.799	1	0.371	0.920	1	0.338
Eigenvector Centrality-by-Environment	49.699	4	**<0.001**	12.803	4	0.012*^	2.269	4	0.687	0.394	4	0.983
Eigenvector Centrality-by-P:C Ratio	6.678	1	**<0.010**	4.330	1	0.037^	0.732	1	0.392	0.023	1	0.880
Eigenvector Centrality-by-Caloric Concentration	9.859	1	**0.002**	10.628	1	**0.001**	1.052	1	0.305	0.188	1	0.664

Supplementary Table 3 | Model results for effects on fitness components. The significance threshold for models including network data was Bonferroni adjusted to account for multiple testing of the five network position variables. Uncorrected p-values are reported. Bolded p-values indicate significant effects, and p-values marked with * indicate marginally significant effects, after adjusting for multiple testing if applicable. P-values marked with ^ or $^{+}$ indicate significant and marginally significant effects, respectively, without multiple testing correction. Source data are provided as a Source Data file.

SUPPLEMENTARY TABLE 4

Nutritional Environments	Number of Social Groups with Networks from Only Day 1	Number of Social Groups with Networks from Only Day 2	Number of Social Groups with Networks from Both Days 1 & 2	Sum by Nutritional Environment
1:1 P:C / 4x Concentration	4	2	5	16
1:2 P:C / 4x Concentration	5	3	2	12
1:4 P:C / 4x Concentration	2	1	5	13
1:4 P:C / 2x Concentration	6	2	1	10
1:4 P:C / 1x Concentration	5	0	0	5
Sum by Day	22	8	13	Total Networks 56

Supplementary Table 4 | Network sample size summary. This table summarizes the number of networks analyzed by each nutritional environment (rows), as well as whether the networks were generated from videos of social groups taken 1, 2, or both days after social groups were established (columns). Forty-three independent social groups had their network structure analyzed, 13 of which had replicate measures of network structure taken across both days of videoing. Source data are provided as a Source Data file.
