# Supplementary material for: Selection on heritable social network positions is context-dependent in Drosophila melanogaster
Source: Nat Commun. 2021 Jun 7;12:3357. doi: 10.1038/s41467-021-23672-1 (PMC8185000; doi:10.1038/s41467-021-23672-1)
Supplement: Supplementary file 3 — Reporting Summary [file 41467_2021_23672_MOESM3_ESM.pdf]

nature research

Corresponding author(s): Eric Wesley Wice

Last updated by author(s): March 30, 2021

Reporting Summary

Nature Research wishes to improve the reproducibility of the work that we publish. This form provides structure for consistency and transparency in reporting. For further information on Nature Research policies, see our Editorial Policies and the Editorial Policy Checklist.

Statistics

For all statistical analyses, confirm that the following items are present in the figure legend, table legend, main text, or Methods section.

n/a	Confirmed	
☐	☒	The exact sample size (*n*) for each experimental group/condition, given as a discrete number and unit of measurement
☐	☒	A statement on whether measurements were taken from distinct samples or whether the same sample was measured repeatedly
☐	☒	The statistical test(s) used AND whether they are one- or two-sided<br>*Only common tests should be described solely by name; describe more complex techniques in the Methods section.*
☐	☒	A description of all covariates tested
☐	☒	A description of any assumptions or corrections, such as tests of normality and adjustment for multiple comparisons
☐	☒	A full description of the statistical parameters including central tendency (e.g. means) or other basic estimates (e.g. regression coefficient) AND variation (e.g. standard deviation) or associated estimates of uncertainty (e.g. confidence intervals)
☐	☒	For null hypothesis testing, the test statistic (e.g. *F*, *t*, *r*) with confidence intervals, effect sizes, degrees of freedom and *P* value noted<br>*Give P values as exact values whenever suitable.*
☒	☐	For Bayesian analysis, information on the choice of priors and Markov chain Monte Carlo settings
☐	☒	For hierarchical and complex designs, identification of the appropriate level for tests and full reporting of outcomes
☐	☒	Estimates of effect sizes (e.g. Cohen's *d*, Pearson's *r*), indicating how they were calculated

Our web collection on statistics for biologists contains articles on many of the points above.

Software and code

Policy information about availability of computer code

Data collection	Fly tracking data was generated using the open source program Caltech FlyTracker 1.0.5. Data curation was conducted using R 3.6.2 and package igraph 1.2.4.2. Source data and code for data compilation is publicly available on Zenodo repository (DOI: 10.5281/zenodo.4642991).
Data analysis	Data analyses were conducted using R 3.6.2; and packages lme4 1.1, DHARMa 0.2.7, glmmTMB, and coxme 2.2. Code for analyses is publicly available on Zenodo repository (DOI: 10.5281/zenodo.4642991).

For manuscripts utilizing custom algorithms or software that are central to the research but not yet described in published literature, software must be made available to editors and reviewers. We strongly encourage code deposition in a community repository (e.g. GitHub). See the Nature Research guidelines for submitting code & software for further information.

Data

Policy information about availability of data

All manuscripts must include a data availability statement. This statement should provide the following information, where applicable:

- Accession codes, unique identifiers, or web links for publicly available datasets
- A list of figures that have associated raw data
- A description of any restrictions on data availability

All data and code used to curate data and conduct analyses is available on Zenodo repository (DOI: 10.5281/zenodo.4642991).

April 2020

Field-specific reporting

Please select the one below that is the best fit for your research. If you are not sure, read the appropriate sections before making your selection.

☐ Life sciences ☐ Behavioural & social sciences ☒ Ecological, evolutionary & environmental sciences

For a reference copy of the document with all sections, see nature.com/documents/nr-reporting-summary-flat.pdf

Ecological, evolutionary & environmental sciences study design

All studies must disclose on these points even when the disclosure is negative.

Study description	Replicate social groups of Drosophila melanogaster flies, where each group consisted of 20 unrelated heterozygous genotypes derived from the Drosophila Genetic Reference Panel, were placed on one of five nutritional environments. Social groups were video recorded, tracked using tracking software, and social networks of pairwise interactions were generated. Four commonly studied fitness measures were also taken of individuals within social groups. Using GLMMs and null models generated via permutation tests, we tested for effects of genotype, nutritional environment, and their interaction on multiple social network position metrics. We further tested how genotype, nutritional environment, network position metrics, and network position-by-nutritional environment interactions affected fitness components.
Research sample	We created 98 replicate social groups of flies. Each social group consisted of 20 unrelated, heterozygous genotypes bred from the Drosophila Genetic Reference Panel (DGRP) (10 males and 10 females per replicate group). Using replicate heterozygous genotypes allows us to alleviate the potentially deleterious effects of homozygous recessive alleles in inbred lines, and generate individuals representative of naturally occurring variation in the wild. Flies were used, as this system allows us to replicate the genotypes of individuals comprising social groups. Flies were videoed at 3-4 days of age, and females were kept for fitness analyses until death (max. 104 days).
Sampling strategy	We predetermined to create 100 replicate social groups of flies, split between 5 different nutritional environments (~20 replicate social groups per nutritional environment treatment). We ended up creating 98 replicate social groups instead of our original goal of 100. This was due to logistical constraints. No data was analyzed until all data had been completely collected. No statistical method was used to predetermine our sample size.
Data collection	Eric Wice collected and/or oversaw all data collection. Three undergraduates and two lab technicians assisted with data collection. One undergraduate and one high-school student assisted with data validation (ground-truthing automated tracking data against manually collected video data). The data collection procedure consisted of: 1) collecting virgin females of homozygous genotypes, 2) pairing these virgins with males of different homozygous genotypes, 3) allowing heterozygous offspring to develop, 4) collecting virgin heterozygous flies, 5) painting flies for individual identification, 6) allowing for development to sexual maturity, 7) making nutritional environment petri dishes, 8) combining groups of flies onto nutritional environments, 9) data collection of observed matings, 10) video recording social groups for two days using Nikon D3300 cameras, 11) breaking down social groups by discarding males and placing females in individual vials with food, 12) transferring females to a new vial every week until death, 13) counting emerging offspring from females' vials 2 and 3 weeks after they are initially introduced to a vial.
Timing and spatial scale	Network data collection began in February 2017 and proceeded through July 2017, with ~8 social groups being created and videoed per week. We had 4 cameras, and were only able to take videos of 2 groups/day with each camera (thus 8 max social groups per week). This sampling schedule was interrupted only twice when I had to pause data collection to attend conferences. Fitness data collection began at the same time as network data collection (February 2017), and proceeded uninterrupted until the last females from the experiment died as part of the lifespan portion of the experiment (October 2017). All data were collected in the Saltz Laboratory at Rice University.
Data exclusions	Data were excluded from network and fitness analyses if any individuals within a social group died before videos were taken, or if any individuals died before individuals were assessed for our four fitness metrics. This criteria was predetermined and necessary, as our measures of genotypic effects on network position metrics rely on the assumption that social groups are replicated.
Reproducibility	We believe that other researchers could easily reproduce our methodology using the information provided in our manuscript. Over the course of our experiment (~8 months), I noticed no changes in the efficacy of our methodology. All attempts to conduct the experiment were successful, and the experiment did not require repeating.
Randomization	All treatments that flies experienced were randomized throughout the experiment. Four out of five nutritional environments were used for social groups created in any given week, in a round-robin design throughout the experiment. This ensured that any effect of the nutritional environment should not be due to unforeseen block effects of week or time.
Blinding	Network data was collected via videotaping the social groups, and using automated tracking software to track the flies. Thus, all network data is 'blind' to observer biases. For female fitness data, the link between individual females and the nutritional environment they previously experienced was dissociated by referring to females by the color of their paint ID and the social group # they came from. Thus, observers were blind to both the genotype and the nutritional environment females were treated with.

Did the study involve field work? ☐ Yes ☒ No

Reporting for specific materials, systems and methods

We require information from authors about some types of materials, experimental systems and methods used in many studies. Here, indicate whether each material, system or method listed is relevant to your study. If you are not sure if a list item applies to your research, read the appropriate section before selecting a response.

Materials & experimental systems

n/a	Involved in the study
☒	☐ Antibodies
☒	☐ Eukaryotic cell lines
☒	☐ Palaeontology and archaeology
☐	☒ Animals and other organisms
☒	☐ Human research participants
☒	☐ Clinical data
☒	☐ Dual use research of concern

Methods

n/a	Involved in the study
☒	☐ ChIP-seq
☒	☐ Flow cytometry
☒	☐ MRI-based neuroimaging

Animals and other organisms

Policy information about studies involving animals; ARRIVE guidelines recommended for reporting animal research

Laboratory animals	Drosophila melanogaster males and females were derived from the Drosophila Genetic Reference Panel (DGRP). Males used in experiments varied from 0-4 days in age, and females were used from 0 days old until death (max. 104 days).
Wild animals	No wild animals were used in this study.
Field-collected samples	No field collected samples were used in this study.
Ethics oversight	No ethical guidance or approval was required for this study, as our species of study was Drosophila melanogaster flies.

Note that full information on the approval of the study protocol must also be provided in the manuscript.
